# iEnhancer-GAN: A Deep Learning Framework in Combination with Word Embedding and Sequence Generative Adversarial Net to Identify Enhancers and Their Strength

**DOI:** 10.3390/ijms22073589

**Published:** 2021-03-30

**Authors:** Runtao Yang, Feng Wu, Chengjin Zhang, Lina Zhang

**Affiliations:** School of Mechanical, Electrical and Information Engineering, Shandong University, Weihai 264209, China; yrt@sdu.edu.cn (R.Y.); 201816507@mail.sdu.edu.cn (F.W.); cjzhang@sdu.edu.cn (C.Z.)

**Keywords:** enhancer, word embedding, sequence generative adversarial net, convolutional neural network

## Abstract

As critical components of DNA, enhancers can efficiently and specifically manipulate the spatial and temporal regulation of gene transcription. Malfunction or dysregulation of enhancers is implicated in a slew of human pathology. Therefore, identifying enhancers and their strength may provide insights into the molecular mechanisms of gene transcription and facilitate the discovery of candidate drug targets. In this paper, a new enhancer and its strength predictor, iEnhancer-GAN, is proposed based on a deep learning framework in combination with the word embedding and sequence generative adversarial net (Seq-GAN). Considering the relatively small training dataset, the Seq-GAN is designed to generate artificial sequences. Given that each functional element in DNA sequences is analogous to a “word” in linguistics, the word segmentation methods are proposed to divide DNA sequences into “words”, and the skip-gram model is employed to transform the “words” into digital vectors. In view of the powerful ability to extract high-level abstraction features, a convolutional neural network (CNN) architecture is constructed to perform the identification tasks, and the word vectors of DNA sequences are vertically concatenated to form the embedding matrices as the input of the CNN. Experimental results demonstrate the effectiveness of the Seq-GAN to expand the training dataset, the possibility of applying word segmentation methods to extract “words” from DNA sequences, the feasibility of implementing the skip-gram model to encode DNA sequences, and the powerful prediction ability of the CNN. Compared with other state-of-the-art methods on the training dataset and independent test dataset, the proposed method achieves a significantly improved overall performance. It is anticipated that the proposed method has a certain promotion effect on enhancer related fields.

## 1. Introduction

**Figure 1:**
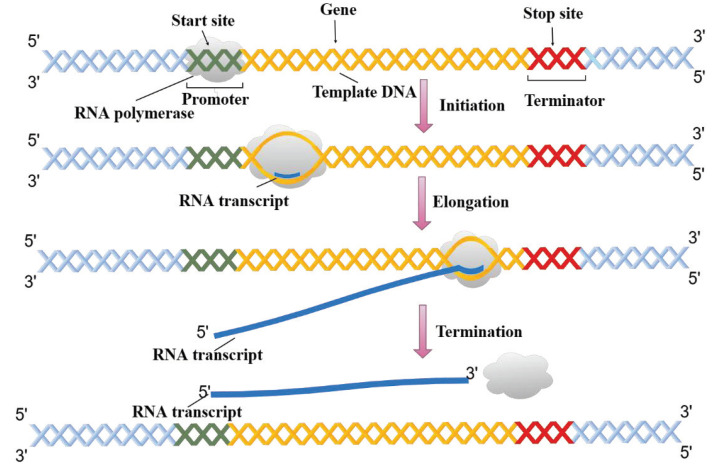
The sub-processes of DNA transcription.

**Figure 2:**
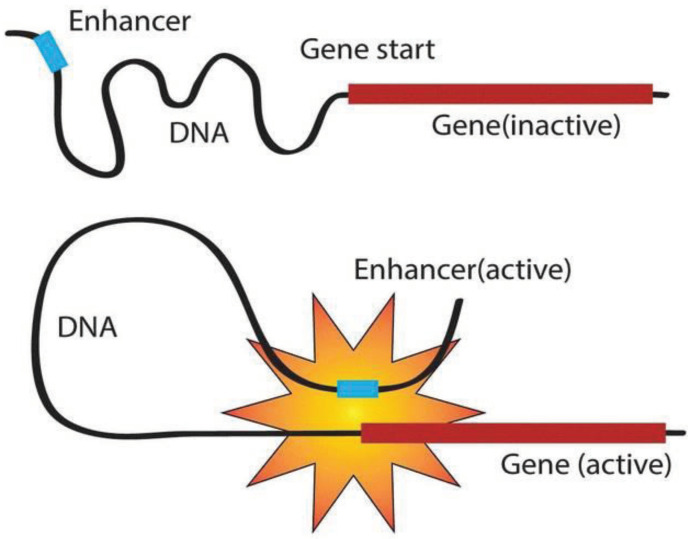
Gene regulations of enhancers.

Expressed as a specific sequence of nucleotides, the genetic information of most living organisms is passed from parent to offspring by DNA replication. During the growth and development of the offspring, the genetic information generally flows from DNA through RNA to proteins via the process of transcription and translation [1]. As shown in Figure 1, transcription can be divided into multiple sub-processes, mainly including initiation, elongation, and termination [2]. Tissue-specific gene transcription is governed by coordinated control of gene-proximal and -distal cis-regulatory elements (CREs) [3]. Among them, as critical components of DNA, enhancers can be commonly bound by transcription factors (TFs) and chromatin modifying enzymes at specific genomic loci to activate gene transcription as given in Figure 2 [4,5]. The regions flanking enhancers are generally characterized by histone modifications and multiple distributed enhancer-promoter interactions [2]. On average, each promoter interacts with 4.9 enhancers, which has an important impact on spatiotemporal gene expression patterns [6]. Recently, advances in epigenomics have demonstrated that enhancer activation and silencing can efficiently and specifically manipulate the biological behavior of downstream genes [7]. Different from classical enhancers in their size, sensitivity of binding to perturbation, and transcription factor density, super-enhancers (SEs) present high potential to maintain cell identity and determine cell fate [8,9]. Notably, the functional role of enhancers on a genome-wide scale remains elusive [6,10]. The comprehensive predictive annotations of enhancers will provide better and more precise insights into the underlining biological roles and molecular mechanisms of enhancers in the spatial and temporal regulation of gene transcription.

Recent studies have linked nucleotide variations in enhancer-associated chromatin-modifying components to a number of phenotypic changes [11,12]. As reported, the absence of a SE can lead to under-expression of cancer associated genes and has profound effects on certain oncogenic properties [13]. Accumulating evidence has underscored that the SEs specific to cancers can induce disordered signaling pathways and contribute to tumor progression [8]. Heyn et al. revealed that SEs undergo abnormal DNA methylation events in cancer development and progression [9]. Cohen et al. indicated that the change of epigenetic characteristics on enhancer elements is an important factor driving the formation of human colorectal cancer [12]. Accordingly, targeting aberrant enhancer components has become an effective therapeutic strategy on various cancers [8,11]. However, tremendous efforts remain to be invested to further clarify the mechanisms underlying enhancer-mediated processes in cancer and other diseases [14,15]. In addition, whether aberrant enhancers drive tumor progression or are merely bystanders in the process of malignant transformation remains unresolved [12]. A more thorough identification of enhancers will potentially facilitate the development of enhancer modulators to overcome trait-associated genetic variants and cancer associated somatic alterations.

For a variety of reasons, profound challenges exist in the identification of novel enhancers [16]. First, enhancer regions are not evolutionarily conserved and account for a very small proportion of the human genome. Second, their positions relative to target genes are flexible and changeable as they do not necessarily interact with the nearest promoter, but can regulate genes located farther away. Third, unlike well-defined protein-coding genes, little is known about the general sequence encoding of enhancers.

With the explosion of genomic and epigenomic data, annotation methods for regulatory elements in specific cell and tissue types have been substantially developed [17]. Traditionally, the ability to regulate transcription detected by reporter gene assays is an important basis for identifying enhancers [2]. Recent advances in next-generation sequencing (NGS) have greatly facilitated the assessment of functional enhancer activity. However, these experimental methods are time-consuming, low-throughput, and applied to limited cell types [6,18]. With this issue in mind, several computational methods have been proposed to identify enhancers and their strength. We focus on machine learning and deep learning approaches for enhancer and its strength identification published from 2016 to 2020. Stimulated by the pseudo amino acid composition, a sequence-based predictor called iEnhancer-2L was proposed with pseudo k-tuple nucleotide composition [19]. Based on support vector machine (SVM), Liu developed a predictor called iEnhancer-PsedeKNC by extracting features from DNA sequences using pseudo degenerate kmer nucleotide composition (PsedeKNC) [20]. Taking bi-profile Bayes and pseudo-nucleotide composition as the feature extraction method, Jia and He employed a two-step wrapper-based feature selection to construct a two-layer predictor called EnhancerPred [21]. Formulating DNA elements with kmer, subsequence profile, and pseudo k-tuple nucleotide composition (PseKNC), Liu et al. developed a new predictor called iEnhancer-EL by key classifiers selected from elementary classifiers [22]. From the angle of the natural language processing, Le et al. combined word embeddings with SVM to develop a novel predictor called iEnhancer-5Step [23]. Taking one-hot encoding and k-mers as the input, Nguyen et al. a convolutional neural network (CNN)-based integrative framework called iEnhancer-ECNN [24]. To develop a predictor called iEnhancer-CNN, Khanal et al. transformed DNA sequences into numerical vectors by word2vec, and then fed them into the CNN [25]. By incorporating multiple features sets, such as k-spectrum profile, mismatch k-tuple, subsequence profile, position-specific scoring matrix (PSSM), and pseudo dinucleotide composition (PseDNC), Cai et al. employed ‘XGBoost’ as a base classifier to construct a two-layer predictor called iEnhancer-XG [26]. Enhancers are generally present in the non-coding region constituted by more than 98% of human genome [16]. More efforts are required for developing computational methods to broadly identify enhancers across the human genome.

The aforementioned methods have obviously facilitated the development of enhancer and its strength identification. However, some limitations still exist. (i) The relative small training dataset with less than 3000 samples is a bottleneck for the performance improvement. (ii) Without considering word frequencies and context information, the word segmentation methods adopted in previous studies have a weak theoretical basis to extract “words” with a fixed-length. In general, for DNA sequences, the functional elements equivalent to the “words” in linguistics vary in length. (iii) Most of machine learning models heavily rely on hand-crafted features, which is generally difficult to automatically extract comprehensive nucleotide patterns from DNA sequences based on the limited domain knowledge, resulting in incomplete DNA representations.

By developing a new predictor in this area, this study is initiated in an attempt to address the limitations as mentioned above. Aiming at the first limitation, the sequence generative adversarial net (Seq-GAN) is employed for data augmentation. Aiming at the second limitation, the word segmentation based on statistics is proposed to incorporate the local and global sequence-order effects without the use of a fixed sliding window. Aiming at the third limitation, the entirely data-driven skip-gram model and the convolutional neural network (CNN) architecture are integrated to automatically mine the hidden high-level discriminative features without involving any manual feature engineering. To the best of our knowledge, the word segmentation based on statistics and the Seq-GAN have not been applied in this research issue.

In conclusion, the main contributions of this paper are as follows. (i) A Seq-GAN model is built to break through the limitations of a small dataset size, thereby improving the quality of the benchmark dataset. (ii) Based on statistics, a word segmentation method is developed to overcome the difficulty in extracting the semantic information of the sequence. (iii) The skip-gram model and the CNN-based deep learning framework are designed to compensate the limitations of traditional machine learning methods in feature construction, thereby improving the performance and robustness of the prediction model.

**Figure 3:**
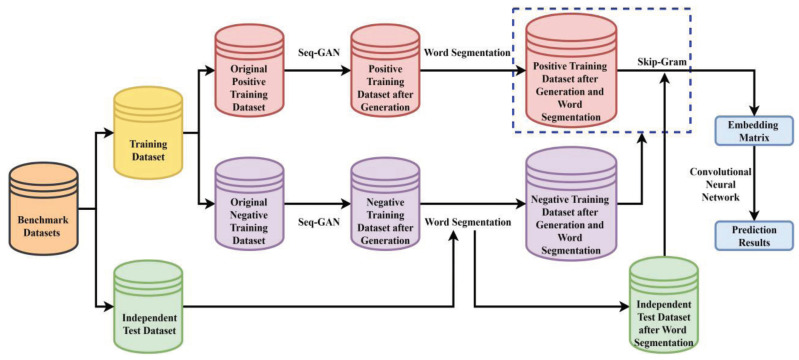
The flowchart of the proposed method iEnhancer-GAN.

The specific processes of the proposed method are implemented as follows. Non-enhancer sequences, strong enhancer sequences, and weak enhancer sequences are, respectively, generated by the Seq-GAN to enlarge the training dataset. DNA sequences are segmented into a series of “words” by the 3-gram word segmentation or word segmentation based on statistics. The skip-gram model is responsible for learning dense feature vectors from these “words” in the positive training dataset to convert DNA sequences into numerical embedding matrices. Finally, a CNN is constructed to extract the hidden high-level discriminative features from these embedding matrices, and then perform the enhancer and its strength identification tasks. The comparison results with existing methods indicate that the proposed method called iEnhancer-GAN achieves a significantly improved overall performance on the training dataset and independent test dataset. The flowchart of the proposed method is illustrated in Figure 3.

## 2. Results and Discussions

### 2.1. Performance Comparisons of Different Word Segmentation Methods

How to extract valid “words” from DNA sequences is particularly important for the performance of a predictive model. The prediction results of word segmentation methods without Seq-GAN on the training dataset and the independent test dataset are, respectively, listed in Table 1 and Table 2. The best performance evaluation indices are highlighted in bold.

For the first layer on the training dataset, the Acc achieved by the word segmentation based on statistics is 0.011 higher than that achieved by the non-overlapped 3-gram word segmentation, and comparable to that achieved by the overlapped 3-gram word segmentation; the Sn obtained by the word segmentation based on statistics is 0.765, which is 0.012 lower than the non-overlapped 3-gram word segmentation and 0.026 lower than the overlapped 3-gram word segmentation; the Sp of the word segmentation based on statistics are significantly higher than that of the other two word segmentation methods; the overlapped 3-gram word segmentation has the largest MCC value of 0.577, only 0.008 higher than the word segmentation based on statistics. In conclusion, these word segmentation methods have similar predictive capabilities for enhancers on the training dataset. For the second layer on the training dataset, the word segmentation based on statistics achieves the highest Sn (0.737), followed by the non-overlapped 3-gram word segmentation (0.715) and the overlapped 3-gram word segmentation (0.714); in terms of Acc and MCC, the word segmentation based on statistics also achieves the best performance; in terms of Sp, the overlapped 3-gram word segmentation scored the highest value of 0.635. Overall, for the enhancer strength identification on the training dataset, the word segmentation based on statistics is better than the other two word segmentation methods.

For the first layer on the independent test dataset, the word segmentation based on statistics achieves the highest Acc of 0.772, Sn of 0.799, Sp of 0.746, and MCC of 0.578. Notably, the MCC of the word segmentation based on statistics is 0.039 higher than that obtained by the overlapped 3-gram word segmentation. For the second layer on the independent test dataset, the Sn and MCC obtained by the word segmentation based on statistics are 0.917 and 0.537, respectively, which are significantly higher than those of the other two word segmentation methods; the non-overlapped 3-gram word segmentation scores the highest Acc of 0.728, which is slightly higher than the word segmentation based on statistics. To sum up, for the identifications of enhancer and its strength on the independent test dataset, the word segmentation based on statistics attains much more outstanding performance, which highlights its excellent generalization ability.

The performance comparisons of word segmentation methods indicate that the word segmentation based on statistics is an ideal choice for DNA sequence segmentations.

### 2.2. Analysis of the Generated DNA Sequences

**Figure 4:**
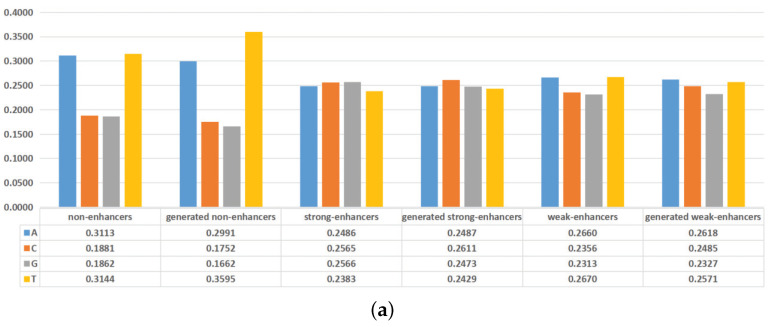

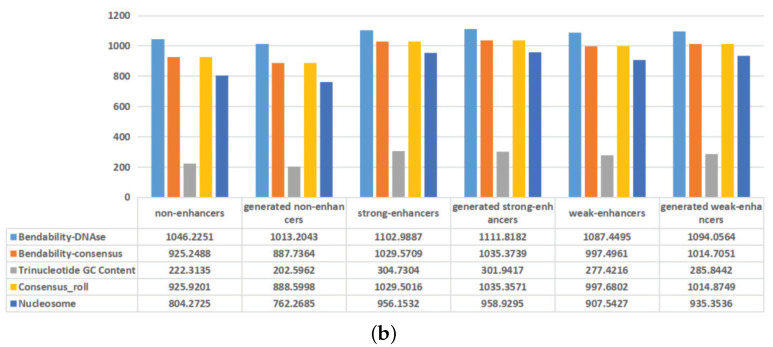
Comparisons between the actual DNA sequences and the generated DNA sequences on nucleotide compositions and mean values of some physicochemical properties. (**a**) The overall frequencies of the nucleotides for the actual DNA sequences and the generated DNA sequences. (**b**) Mean values of some physicochemical properties for the actual DNA sequences and the generated DNA sequences.

In recent years, the nucleotide compositions of DNA sequences have been widely employed to identify functional elements [27,28]. Furthermore, the formation of functional elements is heavily dependent on the physicochemical properties of surrounding nucleotides [29,30]. To intuitively visualize the effectiveness of the generated DNA sequences, the nucleotide compositions and mean values of some physicochemical properties of the actual DNA sequences and the generated DNA sequences are plotted in Figure 4. As shown in Figure 4a, adenine (A) and thymine (T) are preferred to have high frequencies in non-enhancers, while the nucleotides are almost evenly distributed in enhancers. For the actual non-enhancers and the generated enhancers, the frequency of each nucleotide is almost the same. Similar results exist in strong enhancers and weak enhancers. As shown in Figure 4b, the differences between the actual DNA sequences and the generated DNA sequences are rather subtle in terms of mean values of 5 physicochemical properties for trinucleotides. These results indicate that the generated DNA sequences can effectively represent the characteristics of the actual DNA sequences. The inclusion of the generated DNA sequences in the training dataset will highlight the differences of non-enhancers, strong enhancers, and weak enhancers, thus helping to distinguish them.

### 2.3. Effectiveness of the Seq-GAN

The training dataset for enhancer and its strength identification is first enlarged by the Seq-GAN. To intuitively reflect the effectiveness of the Seq-GAN, we list the prediction results with and without the Seq-GAN on the training dataset and the independent test dataset in Table 3 and Table 4, where the word segmentation based on statistics is adopted to extract “words” from DNA sequences.

For the first layer on the training dataset, the performance with the Seq-GAN is superior to that without the Seq-GAN, with the results of Acc, Sn, Sp, and MCC increasing from 0.784, 0.765, 0.803, and 0.569 to 0.951, 0.951, 0.951, and 0.902, respectively. Similar conclusions can be conducted for the second layer on the training dataset. For the first layer on the independent test dataset, all performance measures, except MCC, with the Seq-GAN are superior to those without the Seq-GAN. Similar comparison results can be obtained for the second layer on the independent test dataset. These results demonstrate that the dataset size is indeed important for enhancer and its strength identification. The Seq-GAN can further improve the reliability and performance of the predictor.

### 2.4. Comparisons with Existing Methods

The parameters of the prediction model are adapted to the dataset. In general, the models trained or tested by different datasets will achieve different identification results. To evaluate the prediction performance objectively, we compare our method with previously-published methods on the same training dataset and independent test dataset.

For the proposed method iEnhancer-GAN, the performance measures are calculated by the prediction results of the training dataset rather than the prediction results of the training dataset after data augmentation. Except iEnhancer-GAN, other methods do not consider data augmentation. As listed in Table 5, for the first layer on the training dataset, the prediction performance of iEnhancer-GAN outperforms that of all other methods. There are only 3 methods that provide the Acc over 0.8, while the Acc achieved by iEnhancer-GAN reaches up to 0.951. The Acc, Sn, Sp, and MCC yielded by iEnhancer-GAN are 0.951, 0.951, 0.951, and 0.902, which are 0.128, 0.14, 0.062, and 0.209 higher than the existing best-performing method. Similar results are obtained for the second layer on the training dataset. In conclusion, iEnhancer-GAN exhibits perfect performance for enhancer and its strength identification on the training dataset.

As listed in Table 6, for the first layer on the independent test dataset, in terms of Acc and Sn, iEnhancer-GAN and iEnhancer-5Step achieve pretty close values and outperform all the other methods. iEnhancer-GAN reaches a higher Sp than some existing methods, such as iEnhancer-2L, EnhancerPred, and iEnhancer-XG. The MCC of iEnhancer-GAN is slightly lower than those of iEnhancer-5Step and iEnhancer-CNN, while better than those of all other methods. Overall, iEnhancer-GAN is comparable with iEnhancer-5Step and iEnhancer-CNN, and superior to all the other methods for enhancer identification on the independent test dataset. For the second layer on the independent test dataset, iEnhancer-GAN shows the best performance in terms of Acc, Sn, and MCC. It is worth noting that that the Sn achieved by iEnhancer-GAN is more than 0.2 higher than that achieved by the existing best-performing method, clearly indicating its overwhelming superior for enhancer strength identification.

Except iEnhancer-5step, iEnhancer-ECNN, and iEnhancer-CNN, all the other methods require researchers to carefully design and generate useful features, which is limited by the lack of experiences and domain knowledge. The deep learning framework constructed in the paper can automatically learn expert-free features without involving complex feature extraction and feature selection methods. The word segmentation methods used in iEnhancer-5step, iEnhancer-ECNN, and iEnhancer-CNN are all based on the *n*-gram theory. The biological words are generated by changing the size of *n*. These methods only take the short or local DNA sequence information into account, and ignore the facts that the long or global DNA sequence information is important for the prediction of functional element. Based on statistical theory, the word segmentation method adopted in the paper may incorporate more potential local and global discriminatory information. Except iEnhancer-ECNN and iEnhancer-CNN, all the other existing approaches employs the traditional machine learning algorithms. For iEnhancer-ECNN and iEnhancer-CNN, the size of dataset used for training is equal to 2968. It is far less than normal requirement of deep learning model. The insufficient training data is the potential factor that may cause overfitting. To solve this problem, the Seq-GAN is employed in the paper for data augmentation.

## 3. Materials and Methods

### 3.1. Benchmark Datasets

The benchmark datasets used in this paper for performance analysis and comparison are divided into two parts: training dataset and independent testing dataset.

The benchmark dataset constructed by Liu et al. [19] is employed to train the predictive model. The same dataset was also used in the development of iEnhancer-PsedeKNC [20], EnhancerPred [21], iEnhancer-EL [22], iEnhancer-5Step [23], iEnhancer-ECNN [24], iEnhancer-CNN [25], and iEnhancer-XG [26], which provides a platform to make a fair comparison with previous studies. According to the information on the chromatin state of nine cell lines, the benchmark dataset was constructed by extracting DNA fragments with the length of 200 bp and removing fragment pairs with sequence identity greater than 20%. After randomly selecting non-enhancers and selecting weak enhancers based on the human embryonic stem cell, the training dataset includes 1484 enhancers (742 strong enhancers and 742 weak enhancers) and 1484 non-enhancers.

In order to evaluate the generalization performance of the proposed method, the independent test dataset first collected by Liu et al. [22] is also adopted in this study. It includes 200 enhancers (100 strong enhancers and 100 weak enhancers) and 200 non-enhancers.

The enhancers (positive samples) and non-enhancers (negative samples) are used to train and test the enhancer predictor in the first layer, while the strong enhancers (positive samples) and weak enhancers (negative samples) are used to train and test the enhancer strength predictor in the second layer.

### 3.2. Word Segmentations of DNA Sequences

#### 3.2.1. 3-Gram Word Segmentation

To establish the correspondence between natural language and biological language, DNA sequence is regarded as “sentence”, and it is composed of four kinds of “characters”, Adenine(A), Cytosine(C), Guanine(G), and Thymine(T). Theoretically, A, C, G, and T can also be, respectively, regarded as a “word”. However, only using the four words to depict a long DNA sequence may not completely reflect the inner meaning. For this reason, the *n*-gram word segmentation method is introduced to define the “word”.

According to the central dogma of molecular biology, the genetic codon is composed of 3 consecutive nucleotides of mRNA. It transmits genetic information from mRNA to protein, as well as determines the start, extension, and termination of protein synthesis [31]. In view of this, the 3-gram word segmentation method described in detail below is adopted in this study to define “words” and “dictionary”.

**Figure 5:**
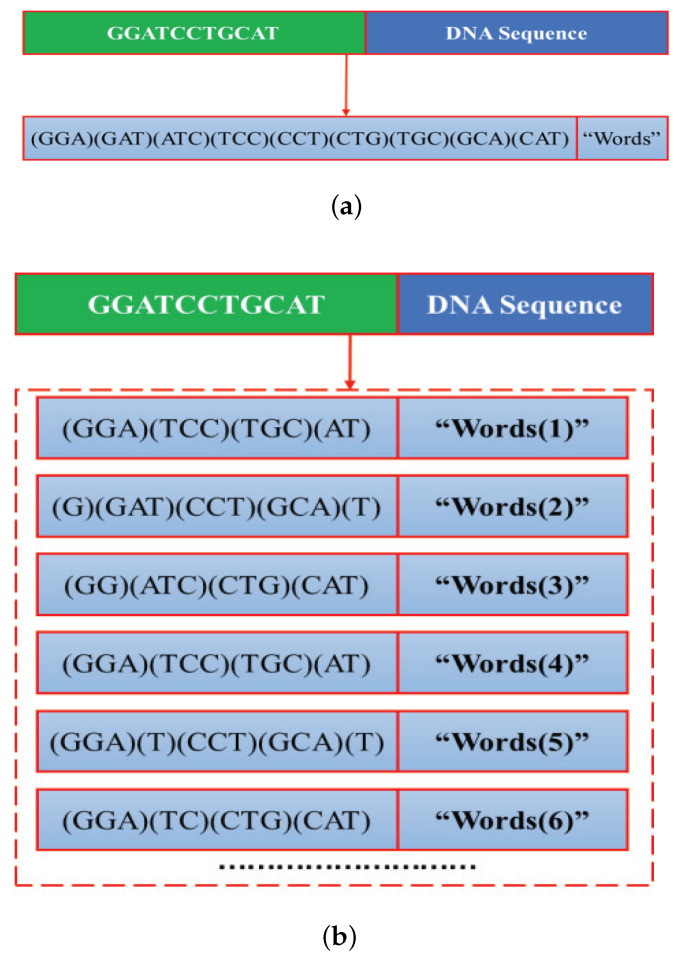
3-gram word segmentation. The DNA subsequence in the bracket represent a “word”. (**a**) Overlapped 3-gram word segmentation. (**b**) Non-overlapped 3-gram word segmentation.

For the overlapped 3-gram word segmentation, as shown in Figure 5a, the “words” are defined as 3 consecutive nucleotides in the DNA sequence, while the “dictionary” is defined as the set of all possible DNA subsequences of length 3.

For the non-overlapped 3-gram word segmentation, as shown in Figure 5b, the “words(i)” is obtained by moving with the window and stride size set as 3 from the *i*-th positions of the DNA sequence. As can be obviously seen from Figure 5b, the “words(1)” and “words(4)” are exactly the same. The differences between the “words(2)” and “words(5)” are only reflected on the first and second word. Similar conclusion can be obtained for the “words(3)” and “words(6)”. To reduce computational complexity without losing too much information, the DNA sequences are segmented into the first three completely different “words”. The “dictionary” consists of 4 mononucleotides, 4×4=16 dinucleotides, and $4^{3}=64$ trinucleotides.

#### 3.2.2. Word Segmentation Based on Statistics

Formally, a character is the smallest unit of a word. In context, the more adjacent characters occur simultaneously, the more likely they are to form a word. Therefore, the co-occurrence frequency or probability of adjacent characters can better reflect/measure the reliability of the word, which is the theoretical basis of the word segmentation based on statistics.

Given a DNA sequence, the word sequence obtained after the word segmentation and the word occurrence probability are, respectively, denoted as $w=\{w_{1},w_{2},\cdots ,w_{m}\}$ and $p(w_{i})$. Assuming that each word is independent of historical words, the word segmentation based on statistics aims to generate the word segmentation $w^{*}$ with the highest probability, which can be formulated as

(1) $$w^{*}=\underset{w}{\arg}\max\prod_{i=1}^{m}p\left(w_{i}\right),w_{i}\in D,$$

where D represents a pre-determined dictionary. In order to obtain enough biological words, the dictionary size is set to 150.

For a long DNA sequence with multiple possible word segmentations, the enumeration method can be used to calculate the probabilities of all word segmentations, but it has a low efficiency. For this reason, this paper takes the possible candidate words as nodes and the probabilities of the word occurrence as the weights of edges to construct a directed segmentation graph. The Viterbi algorithm [32] is employed to find the path with the largest weight as the final word segmentation result.

Obviously, the dictionary D for DNA sequences is ambiguous. An iterative algorithm is adopted in this study to define D through the following steps. (i) The byte-pair-encoding (BPE) [33] algorithm is implemented to establish an initial dictionary by searching the most frequent combinations of nucleotides. (ii) On the basis of a fixed dictionary, $p(w_{i})$ is solved by the expectation maximization (EM) [34] algorithm to maximize the marginal likelihood L in Equation (2), where $X^{i}$ is the *i*-th sequence in the corpus, and its segmentation candidate set is denoted as $S(X^{i})$. (iii) Before and after each word $w_{i}$ is removed, the marginal likelihood $L_{i}$ is, respectively, calculated, and their difference is denoted as $loss_{i}$. (iv) The words are ranked by $loss_{i}$, and the top 70% are retained. (v) Repeat (ii)–(iv) until D meets the desired size.

(2) $$L=\sum_{i=1}^{n}\log\left(p\left(X^{i}\right)\right)=\sum_{i=1}^{n}\log\sum_{w\in S(X^{i})}p\left(w\right).$$

### 3.3. Skip-Gram Model Based on Negative Sampling

Word2vec is a shallow neural network probabilistic language model that can learn word embeddings in an unsupervised manner. It overcomes the problem of high dimensionality and sparseness of word vectors brought by one-hot encoding, and incorporates context information into the word vector representation [35]. As a classical model of word2vec, the skip-gram model [36] predicting context words given a center word is adopted in this study to convert the “words” into numerical vectors.

**Figure 6:**
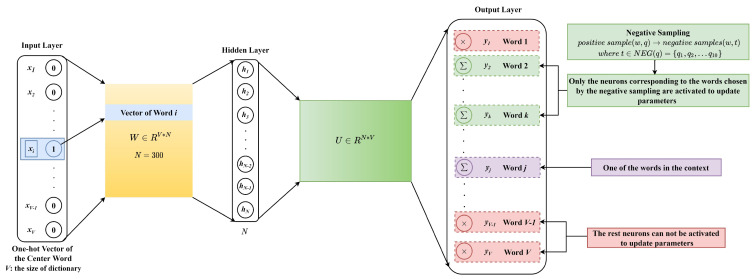
The skip-gram model based on negative sampling.

As shown in Figure 6, the input of the skip-gram model, $x\in R^{1*V}$, is the one-hot representation of the center word. For the overlapped 3-gram word segmentation, V=64; for the non-overlapped 3-gram word segmentation, V=84; for the word segmentation based on statistics, V=150. After passing through a hidden layer with N=300 neurons, the input is mapped to a lower dimensional space. The output of the hidden layer is h=xW, where $W\in R^{V*N}$, and its *j*-th row corresponds to the low-dimensional vector representation of the word labeled as *j*. As *x* is a one-hot vector, xW is a row of *W*, and $h\in R^{1*N}$ is the vector representation of the center word. The output of the model y=hU, where $U\in R^{N*V}$, and its *j*-th column corresponds to the context vector representation of the word labeled as *j*. Based on the Softmax activation function, the predicted probability of the word labeled as *j* is

(3) $$P_{j}=\frac{\exp(h\cdot U_{j})}{\sum_{k=1}^{V}\exp\left(h\cdot U_{k}\right)}.$$

Unlike the update of all weights for each training sample, negative sampling allows only a small part of the weights to be updated at a time, thereby reducing the computational complexity in the gradient descent process. In the skip-gram model, the center word and its context are, respectively, denoted as *w* and context(w). Each word *q* in context(w) and *w* can constitute a positive sample. The number of words in the context is set to 4. Negative sampling is performed for each positive sample (w,q) to obtain m=10 negative samples (w,t), where $t\in NEG\left(q\right)=\left\{q_{1},q_{2},\cdots ,q_{m}\right\}$. For positive samples, the output of the skip-gram model is ${\hat{p}}_{q}=\sigma \left(h\theta^{q}\right)$, where σ is the sigmoid function, and $\theta^{q}$ is the context vector corresponding to the word *q* in the weight matrix *U*. For negative samples, the output of the skip-gram model is ${\hat{p}}_{t}=\sigma \left(h\theta^{t}\right)$. Under this strategy, the optimization goal is to simultaneously maximize the probability of positive samples and minimize the probability of negative samples, that is to maximize

(4) $$F_{w}=\prod_{q\in context(w)}\left[{\hat{p}}_{q}\prod_{t\in NEG(q)}\left(1-{\hat{p}}_{t}\right)\right].$$

For a given corpus C, the final optimization goal is

(5) $$F=\prod_{w\in C}F_{w}.$$

For calculation convenience, take the logarithm of F; then,

(6) $$F=\log\prod_{w\in C}F_{w}=\sum_{w\in C}\log F_{w}=\sum_{w\in C}\sum_{q\in context(w)}\left[\log\left(\sigma \left(h\theta^{q}\right)\right)+\sum_{t\in NEG(q)}\log\left(1-\sigma \left(h\theta^{t}\right)\right)\right].$$

From 1−σ(x)=σ(−x), we can get

(7) $$F=\sum_{w\in C}\sum_{q\in context(w)}\left[\log\left(\sigma \left(h\theta^{q}\right)\right)+\sum_{t\in NEG(q)}\log\left(\sigma \left(-h\theta^{t}\right)\right)\right].$$

The stochastic gradient ascent method [37] is adopted in this study to solve the optimization problem.

Based on the skip-gram model and word segmentation methods described above, the word vectors of DNA sequences can be obtained. If they are directly summed and averaged or horizontally concatenated, each sequence will be represented by a one-dimensional vector, which can be used as the input of traditional machine learning algorithms to achieve classification. However, it may cause the loss of the sequence-order information and the position dependency effects. The sequence personalities is likely to be discarded and converted into commonality. Its efficiency is still hampered by their inability to extract useful features from a robust and automatic framework. In view of this, this study vertically concatenates the word vectors of DNA sequences so that each sequence is represented by a two-dimensional pre-trained embedding matrix.

### 3.4. Construction of Convolutional Neural Network

In recent years, deep learning has promoted the explosive development of artificial intelligence. From the perspective of bionics, deep learning extracts features at different levels, avoiding the explicit feature reconstruction process in traditional machine learning algorithms. Various models of deep learning have fully energized the field of protein functional annotation. To mine remote interaction information, Kaleel et al. proposed a protein relative solvent accessibility prediction framework by combining a bidirectional recurrent neural network with a stack of convolutional layers [38]. By integrating local context and global sequence features, Zeng et al. constructed a new end-to-end deep learning framework to predict protein interaction sites [39].

**Figure 7:**
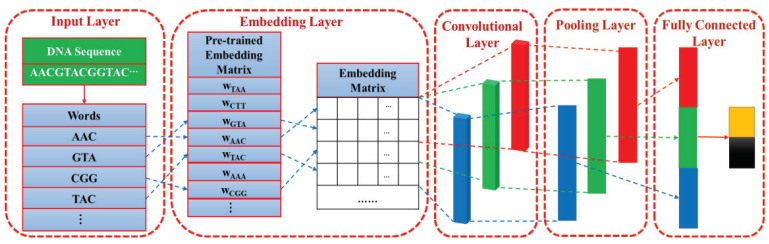
The architecture of the proposed convolutional neural network (CNN).

Convolutional neural network (CNN) is the most typical deep learning architecture. Inspired by visual neuroscience, the essence of CNN is to learn multiple filters that can extract features of input data. As shown in Figure 7, the CNN is composed of input layer, embedding layer, convolutional layer, pooling layer, and fully connected layer, which will be described in detail below.

*Input layer.* In the input layer, a given DNA sequence is segmented into vertically arranged “words” so that each “word” is regarded as an operating unit rather than the entire sequence.

*Embedding layer.* As shown in Figure 7, the construction process of the embedding layer can be viewed as a “query" process, or it can be represented by a fully connected layer. According to the word order of the DNA sequence, the pre-trained word vector $w_{word}$ is added to the corresponding row of the embedding matrix by matching each word with each row index in the pre-trained embedding matrix.

*Convolutional layer.* The convolution operation with a stride of 1 can be formulated as

(8) $$z_{i}^{j}=\sigma \left(sum\left(W_{j}⊙x_{i:i+m-1}\right)+b_{j}\right),$$

where $z_{i}^{j}$ represents the output of the *i*-th local region after passing the *j*-th convolution kernel. It is worth mentioning that the weights are shared when the same convolution kernel is applied to different local regions. σ denotes the rectified linear unit (ReLU) activation function. ⊙ denotes the element-wise product between matrices. sum(A) refers to the sum of all the elements of *A*. $x_{i:i+m-1}$ is a submatrix composed of the first row to the i+m−1-th row of *x*, and *x* represents the embedding matrix of the input DNA sequence. $W_{j}$ and $b_{j}$ are, respectively, the weight and bias of the *j*-th convolution kernel. The size of the convolution kernel is set to 2 × 300, 3 × 300, and 4 × 300, respectively. Each convolution kernel is intended to learn a specific type of feature, and multiple convolution kernels may help in providing insights into different potentially important DNA patterns. The number of convolution kernels with each size is set to 128. Finally, 128 × 3 feature maps are generated in the convolutional layer.

*Pooling layer.* To highlight the key features in the feature map and prevent over-fitting during the training phase, max pooling is performed to extract the maximum value of each feature map.

*Fully connected layer.* The feature vectors generated in the pooling layer are concatenated to form a new feature vector with a dimension of 128 × 3 = 384. Each element of new feature vector is fully connected to 384 neurons, and the Softmax function is used to output the category probabilities.

### 3.5. Sequence Generative Adversarial Net

The CNN-based deep learning model trained by the dataset with less than 3000 samples is prone to over-fitting. To reduce the influence of the problem, the sequence generative adversarial net (Seq-GAN) [40] is employed to rebuild the dataset by generating artificial sequences.

**Figure 8:**
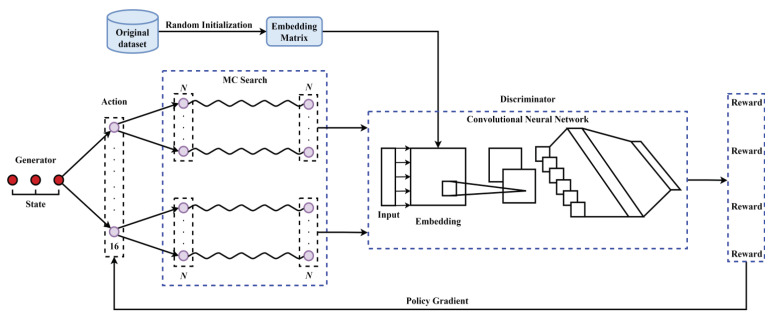
The architecture of the sequence generative adversarial net.

As shown in Figure 8, the Seq-GAN mainly includes a generator and a discriminator. The role of the discriminator is to distinguish real data from generated data, while the role of the generator is to improve itself to generate data that can confuse the discriminator. According to the policy gradient algorithm, the optimization goal of the generator is to maximize the accumulated expected value of reward from the initial state $s_{0}$, which can be formulated as

(9) $$\max J\left(\theta \right)=E\left[R_{T}|s_{0},\theta \right]=\sum_{y_{1}\in Y}G_{\theta }\left(y_{1}|s_{0}\right)\cdot Q_{D\phi }^{G_{\theta }}\left(s_{0},y_{1}\right),$$

where $R_{T}$ is the reward for the complete sequence. θ and ϕ are, respectively, the model parameters of the generator and the discriminator. $y_{t}$ is the output of the generator at time *t*. *Y* is the set of all possible outputs. $Q_{D\phi }^{G_{\theta }}\left(s,a\right)$ is the action-value function, which means that the action *a* is selected under the state *s*, and then the decision is made in accordance with the policy. Taking the output of the discriminator as the reward, $Q_{D\phi }^{G_{\theta }}\left(s,a\right)$ is then defined as

(10) $$Q_{D\phi }^{G_{\theta }}\left(s=Y_{1:t-1},a=y_{t}\right)=\left\{\begin{array}{cc} \frac{1}{N}\sum_{n=1}^{N}D_{\phi }\left(Y_{1:T}^{n}\right),Y_{1:T}^{n}\in MC^{G_{\beta }}\left(Y_{1:t};N\right)\; & for\;t<T\\ D_{\phi }\left(Y_{1:t}\right)\; & for\;t=T \end{array}\right.,$$

where $Y_{1:t}=\left\{y_{1},y_{2},\cdots ,y_{t}\right\}$, and $Y_{t+1:T}^{n}$ is sampled based on the generative model $G_{\beta }$ and the Monte Carlo (MC) search with a roll-out policy.

The generated data is used to retrain the discriminator, and its objective function is

(11) $$\min_{\phi }-E_{R}\left(\log D_{\phi }\left(R\right)\right)-E_{G}\left[\log\left(1-D_{\phi }\left(G\right)\right)\right],$$

where R denotes the real dataset, while G denotes the generated dataset. After training the discriminator for one or more rounds, the generator is updated with the following formula.

(12) θ←θ+α▿J(θ),

where $▿J\left(\theta \right)=\sum_{t=1}^{T}E_{Y_{1:t-1}}\left[\sum_{y_{t}\in Y}▿\theta G_{\theta }\left(y_{t}|Y_{1:t-1}\right)\cdot Q_{D\phi }^{G_{\theta }}\left(Y_{1:t-1},y_{t}\right)\right].$

To ensure that the input format of the Seq-GAN is similar to English sentences, the non-overlapped 2-gram word segmentation is employed for pre-segmentations of DNA sequences. Through the Seq-GAN, 20,000 non-enhancer sequences, 10,000 strong enhancer sequences and 10,000 weak enhancer sequences are, respectively, generated. The generated sequences may have a high similarity with the original sequences. To avoid potential bias and over-fitting, CD-HIT software [41] is used to remove redundant sequences with a cutoff of 80%.

### 3.6. Performance Measures

In this paper, the 10-fold cross-validation [42] is adopted to evaluate the performance of enhancer and its strength predictors. That is, the training dataset is randomly split into 10 disjoint subsets with roughly equal size. Each subset is, in turn, taken as a test set, and the remaining are combined to train the predictor.

The overall prediction accuracy (Acc), sensitivity (Sn), specificity (Sp), and Matthew’s correlation coefficient (MCC) are used to quantitatively measure the prediction performance. They are defined as

(13) $$Acc=\frac{TP+TN}{TP+TN+FP+FN},$$

(14) $$Sn=\frac{TP}{TP+FN},$$

(15) $$Sp=\frac{TN}{TN+FP},$$

(16) $$MCC=\frac{TP\times TN-FP\times FN}{\sqrt{\left(TP+FP)(TP+FN)(TN+FP)(TN+FN\right)}},$$

where *TP*, *FP*, *TN*, and *FN* represent true positives, false positives, true negatives, and false negatives, respectively.

## 4. Conclusions

As critical regulatory elements of DNA, enhancers perform significant roles in gene transcription and are implicated in a series of diseases. Accurately identifying enhancers and their strength could contribute to revealing the underlying mechanisms of enhancer-related biological processes and disease progression. In this study, a promising deep learning framework has been developed to identify enhancers and their strength. Firstly, for the relatively small training dataset, the Seq-GAN is adopted to generate non-enhancers, strong enhancers and weak enhancers. Then, the skip-gram model combined with the word segmentation based on statistics is developed to obtain the embedding matrices, i.e., feature descriptors for DNA sequences. Finally, a CNN architecture is designed to integrate feature extraction and the identification tasks. Experimental results indicate that, compared with the 3-gram word segmentation, the word segmentation based on statistics can extract “words” from DNA sequences more effectively; the actual DNA sequences and the generated DNA sequences have strong similarities on nucleotide compositions and mean values of some physicochemical properties; the performance with the Seq-GAN is superior to that without the Seq-GAN. Furthermore, the proposed method iEnhancer-GAN performs far better than previous methods on the training dataset. On the independent test dataset for the enhancer identification, iEnhancer-GAN is comparable with iEnhancer-5Step and iEnhancer-CNN, and superior to all the other methods; on the independent test dataset for the enhancer strength identification, iEnhancer-GAN shows the best performance in terms of Acc, Sn, and MCC. To further improve the prediction performance, our further work will mainly focus on the ensemble learning techniques and autoencoder-based feature reduction.

## Figures and Tables

**Table 1:** Performance comparisons of different word segmentation methods without Seq-GAN on the training dataset.

Layer	Word Segmentation Method	Acc	Sn	Sp	MCC
First Layer (Enhancer Identification)	Overlapped 3-Gram Word Segmentation	**0.788**	**0.791**	0.786	**0.577**
	Non-Overlapped 3-Gram Word Segmentation	0.773	0.777	0.769	0.546
	Word Segmentation Based on Statistics	0.784	0.765	**0.803**	0.569
Second Layer (Enhancer Strength Identification)	Overlapped 3-Gram Word Segmentation	**0.675**	0.714	**0.635**	0.350
	Non-Overlapped 3-Gram Word Segmentation	0.659	0.715	0.602	0.320
	Word Segmentation Based on Statistics	**0.675**	**0.737**	0.613	**0.353**

**Table 2:** Performance comparisons of different word segmentation methods without Seq-GAN on the independent test dataset.

Layer	Word Segmentation Method	Acc	Sn	Sp	MCC
First Layer (Enhancer Identification)	Overlapped 3-Gram Word Segmentation	0.752	0.781	0.724	0.539
	Non-Overlapped 3-Gram Word Segmentation	0.762	0.784	0.741	0.552
	Word Segmentation Based on Statistics	**0.772**	**0.799**	**0.746**	**0.578**
Second Layer (Enhancer Strength Identification)	Overlapped 3-Gram Word Segmentation	0.718	0.843	**0.593**	0.484
	Non-Overlapped 3-Gram Word Segmentation	**0.728**	0.896	0.560	0.523
	Word Segmentation Based on Statistics	0.724	**0.917**	0.531	**0.537**

**Table 3:** Prediction results with and without Seq-GAN on the training dataset.

Layer	Method	Acc	Sn	Sp	MCC
First Layer (Enhancer Identification)	Without Seq-GAN	0.784	0.765	0.803	0.569
	With Seq-GAN	**0.951**	**0.951**	**0.951**	**0.902**
Second Layer (Enhancer Strength Identification)	Without Seq-GAN	0.675	0.737	0.613	0.353
	With Seq-GAN	**0.872**	**0.873**	**0.871**	**0.744**

**Table 4:** Prediction results with and without Seq-GAN on the independent test dataset.

Layer	Method	Acc	Sn	Sp	MCC
First Layer (Enhancer Identification)	Without Seq-GAN	0.772	0.799	0.746	**0.578**
	With Seq-GAN	**0.784**	**0.811**	**0.758**	0.567
Second Layer (Enhancer Strength Identification)	Without Seq-GAN	0.724	0.917	0.531	**0.537**
	With Seq-GAN	**0.749**	**0.961**	**0.537**	0.505

**Table 5:** The prediction results compared with those of other methods on the training dataset.

Layer	Method	Acc	Sn	Sp	MCC
First Layer (Enhancer Identification)	iEnhancer-2L [19]	0.769	0.781	0.759	0.540
	iEnhancer-PsedeKNC [20]	0.768	0.773	0.763	0.540
	EnhancerPred [21]	0.732	0.726	0.738	0.464
	iEnhancer-EL [22]	0.780	0.757	0.804	0.561
	iEnhancer-5Step [23]	0.823	0.811	0.835	0.650
	iEnhancer-ECNN [24]	0.769	0.785	0.752	0.537
	iEnhancer-CNN [25]	0.806	0.759	0.889	0.693
	iEnhancer-XG [26]	0.811	0.757	0.865	0.627
	iEnhancer-GAN [This Study]	**0.951**	**0.951**	**0.951**	**0.902**
Second Layer (Enhancer Strength Identification)	iEnhancer-2L [19]	0.619	0.622	0.618	0.240
	iEnhancer-PsedeKNC [20]	0.634	0.626	0.644	0.270
	EnhancerPred [21]	0.621	0.627	0.615	0.241
	iEnhancer-EL [22]	0.650	0.690	0.611	0.315
	iEnhancer-5Step [23]	0.681	0.753	0.608	0.370
	iEnhancer-ECNN [24]	0.678	0.791	0.564	0.368
	iEnhancer-CNN [25]	0.764	0.436	0.768	0.451
	iEnhancer-XG [26]	0.667	0.749	0.586	0.340
	iEnhancer-GAN [This Study]	**0.872**	**0.873**	**0.871**	**0.744**

**Table 6:** The prediction results compared with those of other methods on the independent test dataset.

Layer	Method	Acc	Sn	Sp	MCC
First Layer (Enhancer Identification)	iEnhancer-2L [19]	0.730	0.750	0.710	0.460
	EnhancerPred [21]	0.740	0.735	0.745	0.480
	iEnhancer-EL [22]	0.748	0.710	0.785	0.496
	iEnhancer-5Step [23]	**0.790**	**0.820**	0.760	0.580
	iEnhancer-CNN [25]	0.775	0.783	**0.790**	**0.585**
	iEnhancer-XG [26]	0.667	0.749	0.586	0.340
	iEnhancer-GAN [This Study]	0.784	0.811	0.758	0.567
Second Layer (Enhancer Strength Identification)	iEnhancer-2L [19]	0.605	0.470	**0.740**	0.218
	EnhancerPred [21]	0.550	0.450	0.650	0.102
	iEnhancer-EL [22]	0.610	0.540	0.680	0.222
	iEnhancer-5Step [23]	0.635	0.740	0.530	0.280
	iEnhancer-CNN [25]	0.750	0.653	0.761	0.323
	iEnhancer-XG [26]	0.667	0.749	0.586	0.340
	iEnhancer-GAN [This Study]	**0.749**	**0.961**	0.537	**0.505**

## Data Availability

The study did not report any data.

## Abbreviations

The following abbreviations are used in this manuscript:

Seq-GAN	Sequence Generative Adversarial Net
CNN	Convolutional Neural Network
CREs	Cis-Regulatory Elements
TFs	Transcription Factors
SEs	Super-Enhancers
NGS	Next-Generation Sequencing
SVM	Support Vector Machine
PsedeKNC	Pseudo Degenerate Kmer Nucleotide Composition
PseKNC	Pseudo K-tuple Nucleotide Composition
PSSM	Position-Specific Scoring Matrix
PseDNC	Pseudo Dinucleotide Composition
BPE	Byte-Pair-Encoding
EM	Expectation Maximization
MC	Monte Carlo
Acc	Accuracy
Sn	Sensitivity
Sp	Specificity
MCC	Matthew’s Correlation Coefficient
